# Comparison of Random Forest, k-Nearest Neighbor, and Support Vector Machine Classifiers for Land Cover Classification Using Sentinel-2 Imagery

**DOI:** 10.3390/s18010018

**Published:** 2017-12-22

**Authors:** Phan Thanh Noi, Martin Kappas

**Affiliations:** 1 Cartography, GIS and Remote Sensing Department, Institute of Geography, University of Göttingen, Goldschmidt Street 5, 37077 Göttingen, Germany; mkappas@gwdg.de; 2 Cartography and Geodesy Department, Land Management Faculty, Vietnam National University of Agriculture, Hanoi 100000, Vietnam

**Keywords:** Sentinel-2, Random Forest (RF), Support Vector Machine (SVM), k-Nearest Neighbor (kNN), classification algorithms, training sample size

## Abstract

In previous classification studies, three non-parametric classifiers, Random Forest (RF), k-Nearest Neighbor (kNN), and Support Vector Machine (SVM), were reported as the foremost classifiers at producing high accuracies. However, only a few studies have compared the performances of these classifiers with different training sample sizes for the same remote sensing images, particularly the Sentinel-2 Multispectral Imager (MSI). In this study, we examined and compared the performances of the RF, kNN, and SVM classifiers for land use/cover classification using Sentinel-2 image data. An area of 30 × 30 km^2^ within the Red River Delta of Vietnam with six land use/cover types was classified using 14 different training sample sizes, including balanced and imbalanced, from 50 to over 1250 pixels/class. All classification results showed a high overall accuracy (OA) ranging from 90% to 95%. Among the three classifiers and 14 sub-datasets, SVM produced the highest OA with the least sensitivity to the training sample sizes, followed consecutively by RF and kNN. In relation to the sample size, all three classifiers showed a similar and high OA (over 93.85%) when the training sample size was large enough, i.e., greater than 750 pixels/class or representing an area of approximately 0.25% of the total study area. The high accuracy was achieved with both imbalanced and balanced datasets.

## 1. Introduction

There is undoubtedly a high demand for land use/cover maps for the monitoring and management of natural resources, development strategies, and global change studies [1,2,3,4]. Land use/cover maps are one of the most important documents that provide information for various applications, such as land use policy development, ecosystem services, urban planning, conservation, agricultural monitoring, and land use/cover dynamic assessment [5,6,7,8,9]. 

Remote sensing satellite images are considered as one of the most important data sources for land use/cover mapping [10] due to their extensive geographical coverage at an efficient cost while providing irreplaceable information on the earth’s surface [11]. Land use/cover maps are usually produced based on remote sensing image classification approaches [12,13,14]. However, the accuracy and processing time of land use/cover maps using remote sensing images is still a challenge to the remote sensing community [15]. 

Sentinel-2 is the latest generation Earth observation mission of the ESA (European Space Agency) designed for land and coastal applications, and it includes the identical Sentinel-2 A and Sentinel-2 B satellites which launched in June 2015 and March 2017, respectively [16]. Sentinel-2 remains active and enhances the mission of Landsat and SPOT (Systeme Probatoire d’Observation de la Terre) [17]. It is a system with a wide-swath, high spatial resolution (10–60 m), temporal resolution (ten days/five days for Sentinel-2 A, B/Sentinel-2 A + B), and multi-spectral (13 spectral bands) capabilities. It has also gained great attention in research due to its free access and global coverage. A wide range of applications have been studied with Sentinel-2 A (and Sentinel-2 simulation data), such as soil moisture mapping [18], mapping urban surface water bodies [19], forest stress monitoring [20], and quantifying above ground biomass [21]. Clevers et al. [22] used Sentinel-2 data for retrieving LAI and leaf and canopy chlorophyll content of potato crops. Particularly, in land use and land cover mapping, the practicality of Sentinel-2 has been tested and showed the high potential of application [23,24]. However, because it is a new type of satellite imagery, there are only a handful of studies using Sentinel-2 for land use/cover mapping, and thus more research is necessary to conduct and evaluate the usefulness of this imagery.

According to Lu and Weng [25], it is not only the imagery appropriateness but also the right choice of classification method that affects the results of land use/cover mapping. In literature, a variety of classification methods have been developed and tested for land use/cover mapping using remote sensing data [26,27,28]. These methods range from unsupervised algorithms (i.e., ISODATA or K-means) to parametric supervised algorithms (i.e., maximum likelihood) and machine learning algorithms such as artificial neural networks (ANN), k-Nearest Neighbors (kNN), decision trees (DT), support vector machines (SVM), and random forest (RF). 

In the last decade, the nonparametric methods (machine learning based algorithms) have gained great attention of remote sensing based applications. To understand the rise of machine learning methods in land use/cover mapping, we searched the number of articles in the ISI Web of Knowledge (Indexes in SCI-EXPANDED and SSCI) with different keywords (“land cover”* AND “classification”* AND “Maximum Likelihood (/Artificial Neural Networks/k-Nearest Neighbors/Decision Trees/Support Vector Machines/Random Forest)” during the timespan from 2007 to 2017. 

**Figure 1:**
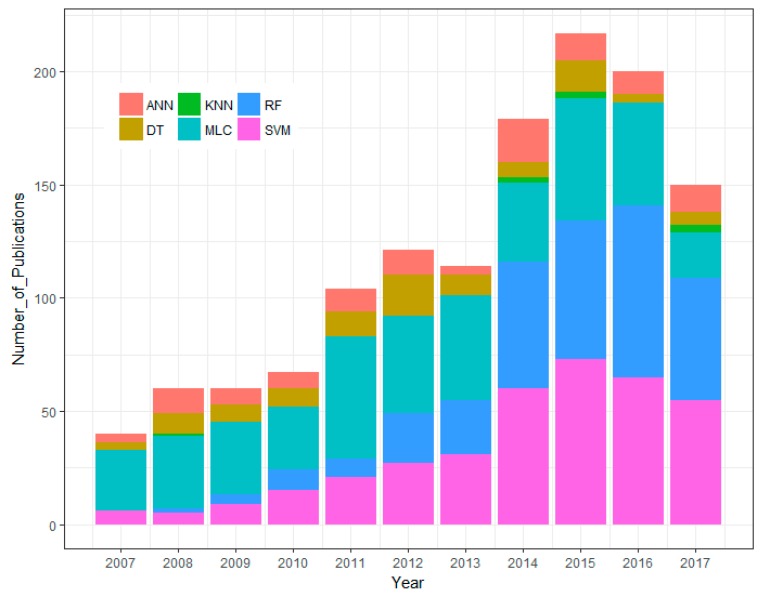
Number of articles in the ISI Web of Knowledge for a general search on different keywords in the last decade (2007–2017*). *Accessed on 13 August 2017.

Figure 1 shows that in this timespan, the use of SVM and RF classification algorithms increased significantly. The number of articles using MLC and ANN have fluctuated throughout the years, but has generally remained steady. In recent years (2014, 2015, and 2017), there were a handful of studies using kNN, however, we have not found any publications using kNN in 2016 with our searched keywords. It should be mentioned that in Figure 1, the high number of papers with the keyword MLC does not mean that there is much research using MLC for classification. In fact, most studies from our searched list used the MLC method as one of the criteria to compare to other machine learning algorithms [11,29,30]. 

The results shown in Figure 1 reflect the studies in recent literature. Prasad et al. [31] stated that DT is too sensitive to small changes in the training dataset. They also reported that DT is occasionally unstable and tends to overfit in the model. The ideal value of k for the kNN classifier is difficult to set [32]. The ANN method contains a high level of complexity in computational processing, causing it to become less popular in remote sensing based classification applications. SVM and RF are insensitive to noise or overtraining, which shows their ability in dealing with unbalanced data [33]. Therefore, among the nonparametric methods, SVM and RF are becoming increasingly popular in image classification studies [34]. 

Several studies have been implemented in order to find the best classification algorithm for land use/cover studies by comparing the performance of these classifiers either among themselves or with other classification algorithms. However, their conclusions are quite different. For example, in the studies by Adam et al. [34] and Ghosh and Joshi. [35], SVM and RF showed similar results of classification. Khatami et al. [11] found that SVM, kNN, and RF generally outperform other traditional supervised classifiers. Pouteau et al. [36] compared 6 machine learning algorithms (SVM, Naïve Bayes, C4.5, RF, Boosted Regression Tree, and kNN) with 6 satellite data sets from different sensors (Landsat-7 ETM+, SPOT, AirSAR, TerraSAR-X, Quickbird, and WorldView-2) for Topical Ecosystems Classification and stated that kNN better performs for the Landsat-7 ETM+ classification. Most recently, Heydari and Mountrakis [37] studied the effects of the classifier selection, reference sample size, reference class distribution, and scene heterogeneity in per-pixel classification accuracy using 26 Landsat sites with five classification algorithms (Naïve Bayesian, kNN, SVM, Tree ensemble, and Artificial Neural Network). They concluded that SVM and kNN were the best classification methods for Landsat classification. 

In addition, to the best of our knowledge, only a limited amount of research was published that compared and evaluated the performance of RF, SVM, and kNN with different training sample strategies using Sentinel-2 imagery, especially in Vietnam. Therefore, it is practical for a study to compare and evaluate the performance of RF, SVM and kNN for land use/cover mapping over North Vietnam using the new satellite data, Sentinel-2 A. The objectives of this study are: (i) to evaluate the performance of the three most increasing classifiers, RF, kNN, and SVM, when applied to a Sentinel-2 image and (ii) to assess the effects of the training samples size, strategies, and type (balanced/imbalanced) on the accuracy of the classification results of the three aforementioned classifiers.

## 2. Materials and Methods 

**Figure 2:**
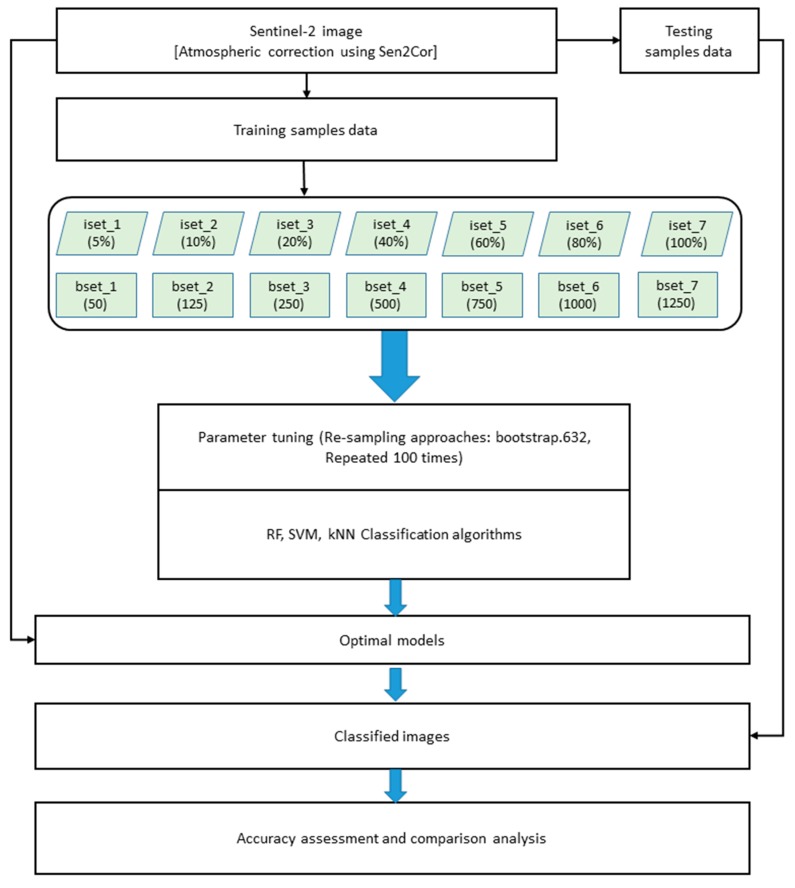
Flowchart of the study methods.

**Figure 3:**
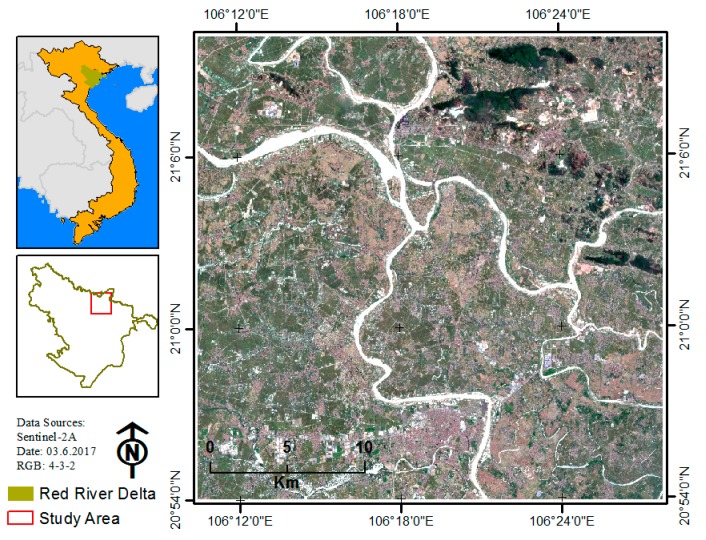
Location of the study area in the north of the Red River Delta (RRD), Vietnam.

The overall methodology of the study is described in Figure 2. To fulfill the study objectives, a study area was selected based on the land cover characteristics and the availability of remote sensing imagery data. The remote sensing image was preprocessed, atmospherically corrected, and clipped to the study area (Figure 3). 

### 2.1. Study Area

In this study, in order to compare the performance of different classification algorithms on different data training sample strategies, an area of 30 × 30 km^2^ of a peri-urban and rural with heterogeneous land cover area in the north of the Red River Delta (RRD), Vietnam was chosen (Figure 3). This is a typical land use/cover of the RRD area, slightly sloping from the southwest to the northeast. The study area mainly includes six typical classes: resident (fragment and distribution over the study area), impervious surface (including factory, block building and transportation, roads), agriculture, bare land, forest, and water. 

### 2.2. Data Used

Sentinel-2 A, level 1 C (ID = L1C_T48QXJ_A010168_20170603T034152) acquired on 3 June 2017, was downloaded from the United States Geological Survey (USGS) website [38]. Spectral bands of the Sentinel-2 A satellite imagery are shown in Table 1. The imagery was atmospherically corrected using the Sen2cor tool, which is available in the Sentinel Application Platform (SNAP) toolbox [22,39]. After atmospheric correction, 10 bands (2–8, 8A, 11 and 12) were composited with 20 m resolution and clipped to the study area (30 × 30 km^2^). The composited-10 band-20m imagery was used for classification in this study. 

### 2.3. Training and Testing Sample Datasets

The training data (training and testing samples) was collected based on the manual interpretation of the original Sentinel-2 data and high-resolution imagery available from Google Earth.

To collect training sample data, the create polygon tool in the ArcGIS 10.5 toolbox was used to create 135 polygons for each land cover class. Due to the different polygon sizes, the number of pixels for each land cover class also differed (Table 2).

For an accurate assessment of the classification results, 650 points for every land cover class was collected. However, to ensure that the training and testing datasets were independent, we buffered 15 m for all point samples (testing dataset) and removed points which had buffered-points intersecting with (or belonging to) polygon samples. As results, we obtained the number of testing points (pixels) as shown in Table 2. 

To evaluate the effect of the training sample sizes, as well as the performance of classification algorithms on the classification accuracies, we randomly divided training sample data into 14 different datasets (Table 3), in which seven-imbalanced datasets (iset_1, iset_2, iset_3, iset_4, iset_5, iset_6, and iset_7) had the corresponding sizes of 5%, 10%, 20%, 40%, 60%, 80%, and 100% of the total training data. The create Data Partition function in the caret package [40] was used to guarantee that all datasets had the same proportion-training sample of each land cover class. The remaining seven-balanced datasets were created as follows: bset_1 (50 pixels/class), bset_2 (125 pixels/class), bset_3 (250 pixels/class), bset_4 (500 pixels/class), bset_5 (750 pixels/class), bset_6 (1000 pixels/class), and bset_7 (1250 pixels/class). The number of pixels in each class for every sub-dataset was chosen to keep the most consistent size between the imbalanced and balanced training sample size; for example, the lowest number of pixels among the 6 land cover classes was 1267 pixels, leading bset_7 to have 1250 pixels for the balanced data for each class. 

### 2.4. Classification Algorithms and Tuning Parameters

Tuned parameters play an important role in producing high accuracy results when using SVM, RF, and kNN. Each classifier has different tuning steps and tuned parameters. For each classifier, we tested a series of values for the tuning process with the optimal parameters determined based on the highest overall classification accuracy. In this study, the classified results under the optimal parameters of each classifier were used to compare the performance of classifiers [41]. 

#### 2.4.1. Support Vector Machine (SVM) 

In land cover classification studies, according to Knorn et al. [42] and Shi and Yang. [43], the radial basis function (RBF) kernel of the SVM classifier is commonly used and shows a good performance. Therefore, we used the RBF kernel to implement the SVM algorithm. There are two parameters that need to be set when applying the SVM classifier with RBF kernel: the optimum parameters of cost (C) and the kernel width parameter (γ) [41,44]. The C parameter decides the size of misclassification allowed for non-separable training data, which makes the adjustment of the rigidity of training data possible [45]. The kernel width parameter (γ) affects the smoothing of the shape of the class-dividing hyperplane [46]. Larger values of C may lead to an over-fitting model [35], whereas increasing the γ value will affect the shape of the class-dividing hyperplane, which may affect the classification accuracy results [35,47]. Following the study of Li et al. [28] and pretested to our dataset, in this study, to find the optimal parameters for SVM, ten values of C (2^−2^, 2^−1^, 2^0^, 2^1^, 2^2^, 2^3^, 2^4^, 2^5^, 2^6^, 2^7^), and ten values of γ (2^−5^, 2^−4^, 2^−3^, 2^−2^, 2^−1^, 2^0^, 2^1^, 2^2^, 2^3^, 2^4^) were tested. This procedure was applied to all 14 sub-datasets. 

#### 2.4.2. Random Forest (RF) 

In order to implement the RF [33], two parameters need to be set up: the number of trees (ntree) and the number of features in each split (mtry). Several studies have stated that the satisfactory results could be achieved with the default parameters [12,48,49,50]. However, according to Liaw & Wiener [48], the large number of trees will provide a stable result of variable importance. In addition, Breiman [33] stated that using more than the required number of trees may be unnecessary, but this does not harm the model. In addition, Feng et al. [51] stated that with ntree = 200, RF could achieve accurate results. Regarding the mtry parameter, there are many studies that use the default value mtry = √p, where p is the number of predictor variables [12]. However, in this study, to find the optimal RF model for classification, a range of values for both parameters were tested and evaluated: ntree = 100, 200, 500, and 1000; mtry = 1:10 with a step size of 1. 

#### 2.4.3. k-Nearest Neighbor (kNN)

The kNN approach is a non-parametric [52] that has been used in the early 1970’s in statistical applications [53]. The basic theory behind kNN is that in the calibration dataset, it finds a group of k samples that are nearest to unknown samples (e.g., based on distance functions). From these k samples, the label (class) of unknown samples are determined by calculating the average of the response variables (i.e., the class attributes of the k nearest neighbor) [54,55]. As a result, for this classifier, the k plays an important role in the performance of the kNN, i.e., it is the key tuning parameter of kNN [41]. The parameter k was determined using a bootstrap procedure. In this study, we examined k values from 1 to 20 to identify the optimal k value for all training sample sets.

### 2.5. Accuracy Assessment and Comparisons

In order to assess the accuracy of classification performance, there are many metrics available in the literature. The two most popular metrics are overall accuracy (OA) and Kappa statistic. Recently however, the Kappa statistic is becoming less common in remote sensing classification accuracy assessment [37]. One of the drawbacks of solely using the OA metric is that it does not show the specific performance of classes. He and Garcia [56] stated that if input datasets (training samples) are highly imbalanced, the OA value might be deceiving, because the rare classes may be classified very poorly. He and Garcia [56] also suggested that when choosing OA as the criterion metric, the best class distribution is followed by the naturally occurring. 

In this study, we used a stratified sampling approach; moreover, we divided training data into several sub-datasets, including imbalanced and balanced datasets. This approach matches the OA metric. In addition, to compare the performance of each classifier, we used the same training (input) and testing (validation) datasets; thus, the effect of individual class’s distribution on OA does not bias the results. We also calculated the 95% confidence interval (error tolerance) δ of the probability estimate [57] for every OAs. Because we used the same testing datasets for all classification accuracy assessment, thus the δ were not significant different. (The detail of OAs and δ are shown in Appendix B). Therefore, to assess and compare the performance of the classifiers and the different datasets, we used overall accuracy (OA) as the criterion. In total, we have seven imbalanced datasets, seven balanced datasets, and three classifiers. Consequently, each classifier had 14 classification results, totaling 42 overall classification results. 

## 3. Results

### 3.1. The Effects of Tuning Parameters on Classification Accuracies

Due to the limitation of space and the consistency of the results, only the results of eight sub-datasets—4 balanced (bset_1, bset_3, bset_5, bset_7) and 4 imbalanced (iset_1, iset_3, iset_5, iset_7)—are shown. The results of the remaining sub-datasets were still tested and showed a consistent trend in results.

#### 3.1.1. The kNN Classifier 

With the kNN classifier, to classify one object, the algorithm bases the class attributes of its k nearest neighbors [41]. Therefore, the k value plays an important role in the performance of kNN, and is the key tuning parameter of kNN algorithm. In this study, we tested a range of k values (1 to 20) for choosing the optimal parameter of the kNN classifier using different sub-datasets. 

**Figure 4:**
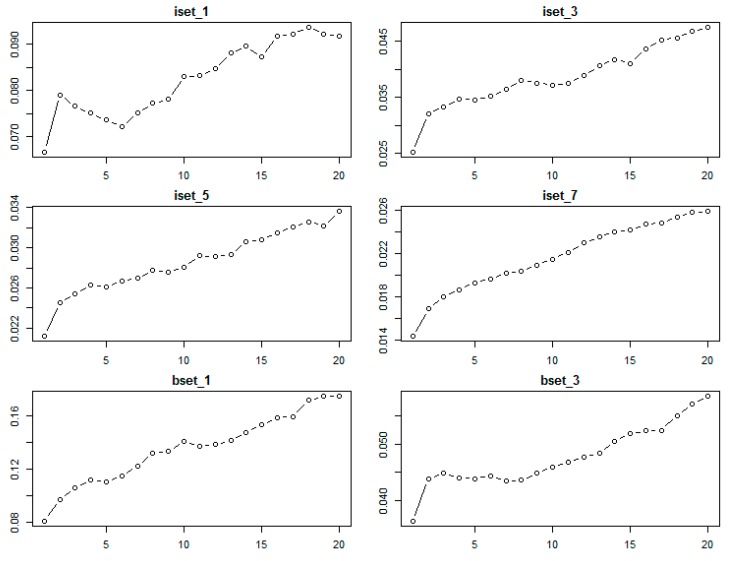

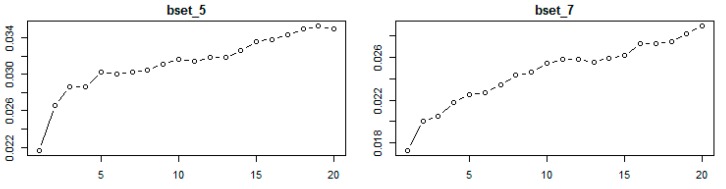
The relationship between classification error (*y*-axis) and k value (*x*-axis) parameters (1 to 20) of the kNN classifier obtained from the bootstrap resampling approach using different sub-datasets of training sample data.

Figure 4 shows the results of the kNN classifier error when applied to different sub-datasets. The lowest error was achieved with k = 1 for all datasets. As shown in Figure 4, when k increases from 1 to 20, the error of the kNN classifier subsequently increases. This finding is consistent with the study by Qian et al. [46]. Therefore, the optimal k for the kNN classifier was chosen as k = 1. 

#### 3.1.2. The RF Classifier

**Figure 5:**
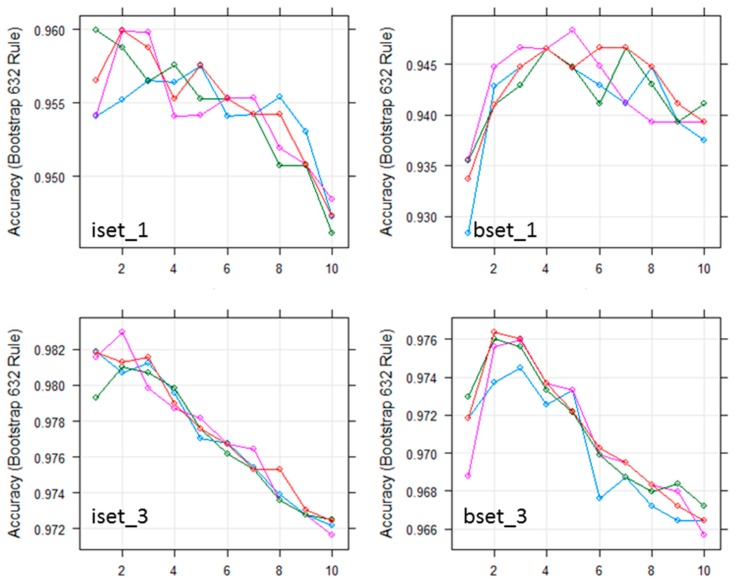

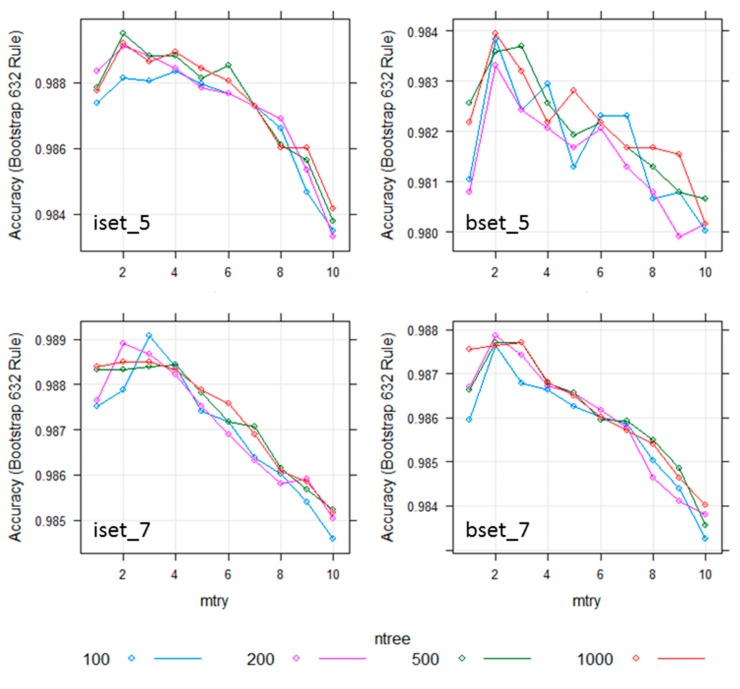
Effect of the number of trees and the number of random split variables at each node (mtry) on the overall accuracy for RF classification using all training sample data.

As stated in Section 2.4.2, there are two parameters that significantly affect the performance of the RF classifier: ntree and mtry. In this study, we used Sentinel-2 with ten bands for classification, meaning the input data has 10 variables. To find the optimal parameters for the RF classifier, several values (mtry = 1:10; ntree = 100, 200, 500, and 1000) were tested for all 14 sub-datasets. The highest results for all sub-datasets were obtained with mtry equal to 2, 3, or 4 (Figure 5). 

**Figure 6:**
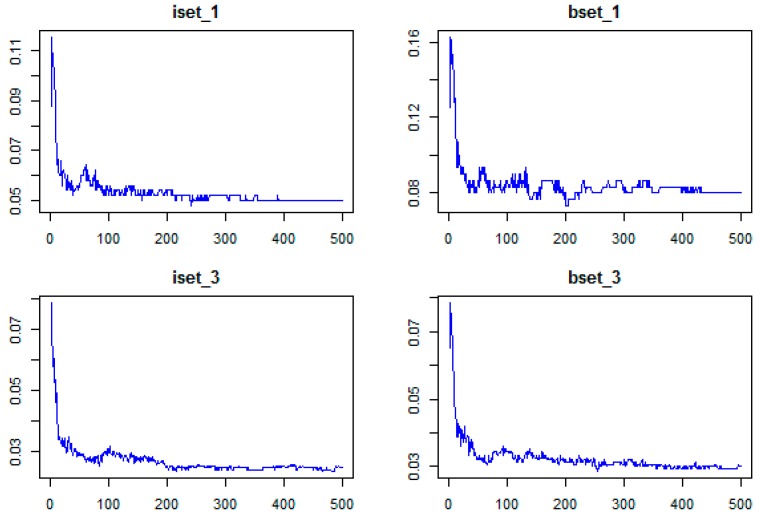

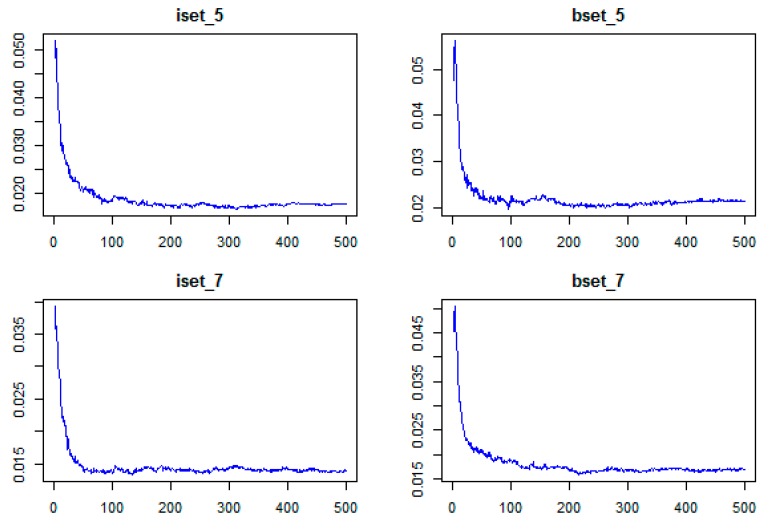
The relationship between OOB error (*y*-axis) and ntree parameter (*x*-axis) of the RF classifier using different sub-datasets of training sample data.

As shown in Figure 5, when the mtry was 2, 3, or 4, the results of ntree 200, 500, and 1000 were similar. In addition, Figure 6 shows that out-of-bag (OOB) error decreased sharply when ntree increased from 1 to 100. When ntree increased from 101 to 400, different sub-datasets had slightly different trends, however, generally, the OOBs were slightly reduced at all sub-datasets. All OOBs of all sub-datasets were almost remain stable when ntree increase from 400 to 500. Therefore, ntree = 500 was used as the optimal value for RF classifiers. The mtry value was chosen based on the highest results of mtry = 2, 3, or 4. 

#### 3.1.3. The SVM Classifier

In order to find the optimal values for the SVM model, several values were examined for C and γ: C (2^−2^, 2^−1^, 2^0^, 2^1^, 2^2^, 2^3^, 2^4^, 2^5^, 2^6^, 2^7^), γ (2^−5^, 2^−4^, 2^−3^, 2^−2^, 2^−1^, 2^0^, 2^1^, 2^2^, 2^3^, 2^4^).

**Figure 7:**
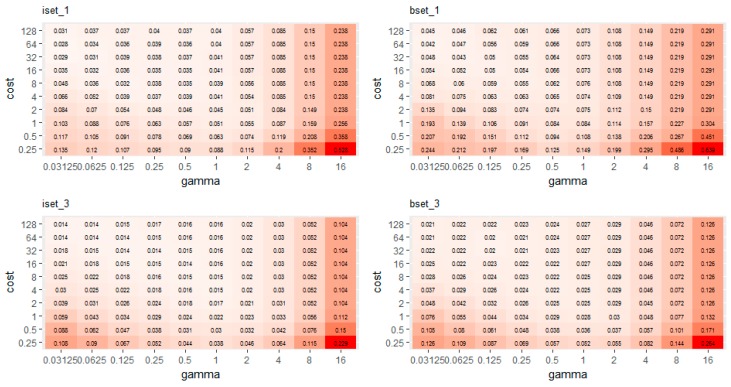

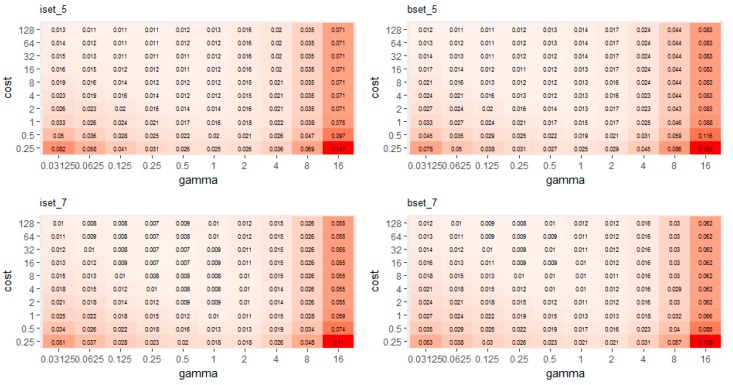
The relationship between classification error and parameters (C and γ) of the SVM classifier obtained from different sub-datasets of training sample data.

Figure 7 shows the relationship between the OOB error and the SVM parameters. Based on these results, the optimal parameters of the 14 sub-datasets were chosen according to the lowest OOB error for each result. In general, a high value of C and a low value of γ produced the lowest error. The higher error was observed with a low C value with both high (greater than eight) and low (less than 0.125) value of γ (Figure 7). The optimal parameters (C and γ) of different sub-datasets varied (Table 4). 

The optimal models (using optimal parameters) were applied to the whole image for classification results. 

### 3.2. The Performance of Different Classifiers on Imbalanced Datasets

**Figure 8:**
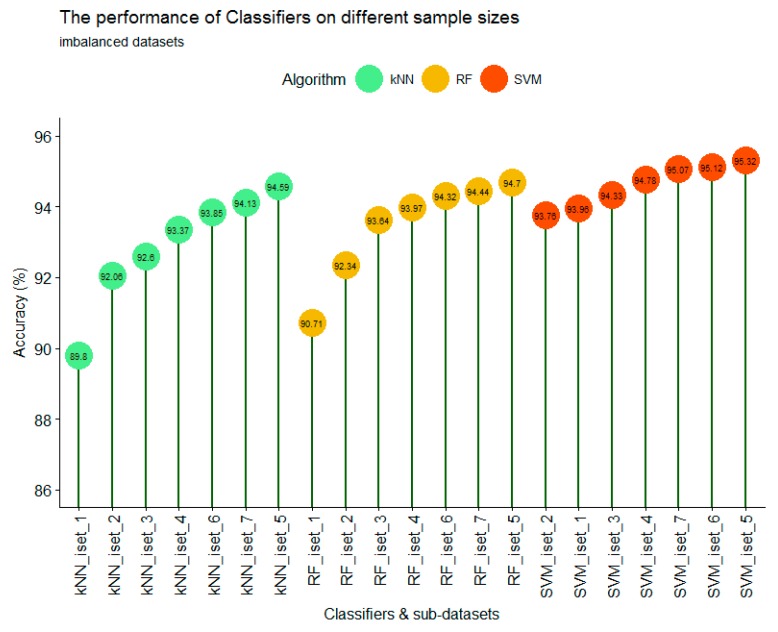
The performance of the kNN, SVM, and RF classifiers on different imbalanced training sample sizes.

As shown in Figure 8, with all seven sub-datasets (iset_1 to iset_7), SVM always showed the most accurate results, followed by RF and kNN. However, the three highest accuracies of all classifiers were only slightly different. The accuracy results of SVM were not significantly different among different training sample sizes; from iset_1 to iset_7, the lowest was iset_1 data at 93.76% and the highest was iset_5 data at 95.32%. In contrast, the classification accuracy of kNN and RF were significantly different between small sample sizes (iset_1) and large sample sizes (iset_7). With small and imbalanced training samples (iset_1, iset_2, and iset_3), there is a difference between classification accuracies. SVM produced a significantly higher accuracy than that of RF and kNN. This is consistent with the results reported from Shi and Yang [43]. From a small sample size (iset_1) to a larger sample size (iset_7), the accuracy significantly increased with RF and kNN, whereas the results of SVM were only slightly increased. It is indicated that the sample size and imbalanced data of training samples has more impact on the classification accuracy for kNN and RF than for SVM. 

The highest accuracy for the three classifiers occurred when the training sample size was large enough, i.e., iset_5; kNN, RF, and SVM were 94.59%, 94.70%, and 95.32%, respectively (Figure 8). However, when the training sample sizes increased further (iset_6 and iset_7), the overall accuracy of the classifiers slightly decreased; kNN, RF, and SVM for iset_6 (iset_7) were 93.85% (94.13%), 94.32% (94.44%), and 95.12% (95.07%), respectively. It is suggested that if the training sample data is imbalanced between classes, the training sample sizes should be large enough to achieve the best performance of classifiers. If the training sample size is too large, it could change the proportion between classes, which lead to the decrease in overall accuracy. For all three classes in this study, the highest accuracies were achieved when the training sample size represented approximately 0.26% of the total study area. This is consistent with research of Colditz [58]; in which they stated that for the RF classifier, the training sample size should account for 0.25% of the total study area. Our results show that this case is not only valid for the RF classifier but also for the SVM and kNN classifiers.

### 3.3. The Performance of Different Classifiers on Balanced Datasets

**Figure 9:**
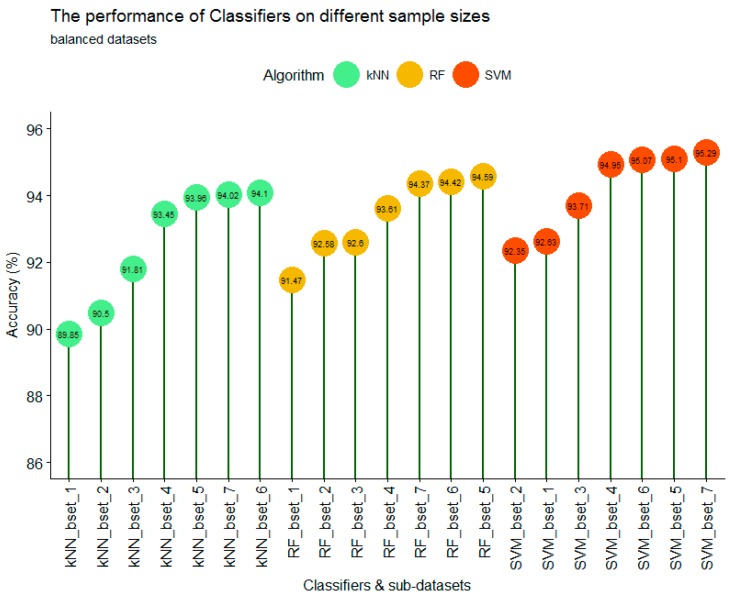
The performance of kNN, SVM, and RF classifiers on different balanced training sample sizes.

For balanced datasets (bset_1 to bset_7), the SVM classifier still produced the highest accuracy at 95.29%, followed by RF at 94.59% and kNN at 94.10%. However, the performance of each classifier on different training sample sizes was only slightly different (Figure 9). For the kNN classifier with a small training sample size (bset_1 to bset_4), the training sample size had a strong impact on the accuracy of classification. The overall trend showed that the larger the training sample size, the higher the accuracy. Increasing the sample size from 50 pixels/class (bset_1) to 500 pixels/class (bset_4) resulted in an accuracy increase from 89.85% to 93.45%. However, when the training sample size was high enough (more than 750 pixels/class), as it is for bset_5, bset_6, and bset_7, the classification accuracy was stable at 93.96%, 94.10%, and 94.02%, respectively. 

With the RF classifier, the larger training sample also produced higher accuracy with the first 4 sub-datasets (bset_1 to bset_4). However, the difference at bset_1 and bset_4 was not as large as with the kNN classifier. With the smallest training sample (bset_1), RF produced a higher accuracy (91.47%) than that of kNN (89.95%); however, with bset_4 (750 pixels/class), the accuracy results of RF and kNN were similar at 93.61% and 93.45%, respectively. The stable accuracy results of bset_5, bset_6, and bset_7 (93.47%, 94.42%, 94.59%) were also observed with the RF classifier. 

The SVM classifier showed different results. When the training sample size was small (bset_1 and bset_2), the classification accuracy was high and slightly different at 92.63% and 92.35%, respectively. However, when the training sample size increased from 125 pixels/class (bset_2) to 250 pixels/class (bset_3) and 500 pixels/class (bset_4), the training sample size had a strong impact on classification accuracy. This is an interesting finding; because the results showed a contrast with previous studies which stated that the training sample size was less sensitive with SVM. This might be true if the training sample size is small enough (less than 125 pixels/class) or large enough (greater than 750 pixels/class). Figure 9 shows that when the balanced training sample size increasing from 0.20% (bset_5) to 0.33% (bset_7) of the total study area, the performance of the classifiers were similar between different training sample sizes. When comparing the three classifiers, SVM produced the highest accuracy, followed by RF and kNN. 

## 4. Discussion

**Figure 10:**
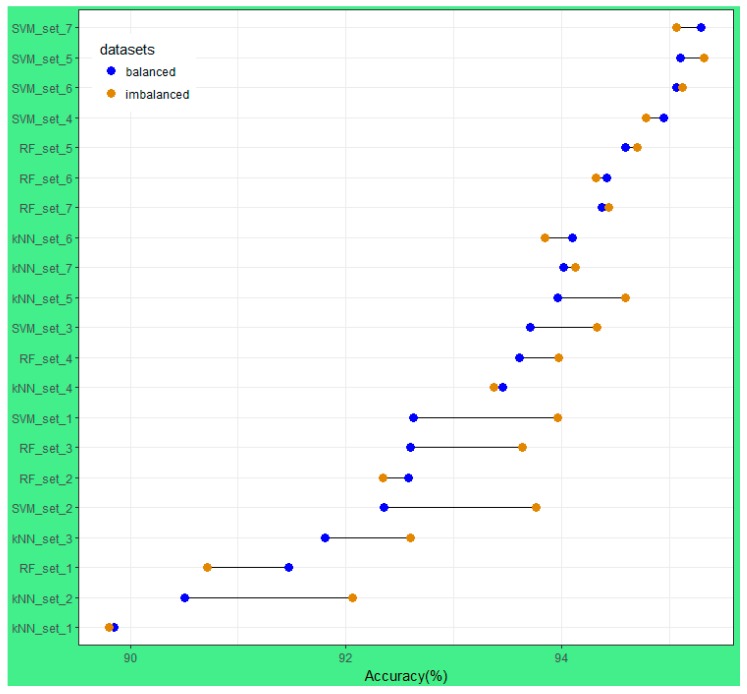
The difference in OA using balanced and imbalanced datasets.

Figure 10 shows the difference between OA of imbalanced and balanced sub-datasets from the 42 results of the 14 different training sample sizes for each classifier. Two different trends are clear: when training sample sizes were large (greater than or equal to 500 pixels/class) the performance of kNN, RF, and SVM on balanced and imbalanced datasets was not significantly different, except for kNN with dataset_5 (approximately 750 pixels/class). 

Due to the actual proportion of land cover type on the landscape, the rare classes have a low number of pixels in the training sample (Table 2); therefore, in all sub-datasets (set_1 to set_7), the number of pixels in balanced datasets is always smaller than those of imbalanced sub-datasets. However, as mentioned earlier, when the training sample size is large enough, the performance of classifiers on balanced and imbalanced sub-datasets was similar. In other words, the classifiers are less sensitive to the imbalanced training data if the training sample size is large enough (i.e., greater than 750 pixels/class). 

Figure 10 shows that the performance of kNN at all sub-datasets for dataset_1, dataset_6, and dataset_7 was similar for balanced and imbalanced datasets, whereas the results of kNN on the remaining datasets were significantly different. It is indicated that the kNN classifier is less sensitive with imbalanced training sample data, although it varies with training sample sizes. With the RF and SVM classifiers, when training sample size is large enough (dataset_5, dataset_6, and dataset_7) the results of imbalanced and balanced were similar and very high (greater than 94.32%). 

Many studies investigated the performance of the RF classifier on different training sample sizes and strategies of different satellite images, but the conclusions are contradictory. Colditz [58] and Mellor et al. [59] found a trend for the area proportional allocation of training samples, in which the greater the land cover class area is the more training samples are needed to produce the best classification accuracy. In contrast, Dalponte et al. [60] and Jin et al. [61] stated that the RF classifier would perform better with balanced training sample data. Our results (Figure 9) show that if training sample size is small (less than 500 pixels/classes) the difference in accuracy of imbalanced and balanced data would be large (dataset-1, dataset-3), or small (dataset-2). However, when the training sample size is large enough (dataset-5, dataset-6, and dataset-7) the performance of RF on balanced and imbalanced training samples were similar. It must be mentioned that for those different conclusions, the RF classifier was used with different satellite imagery. Dalponte et al. [60] used HySpex-VNIR 1600 and HySpex-SWIR 320i data, Jin et al. [61] used Landsat imagery, Colditz [58] used MODIS imagery, and Mellor et al. [59] used Landsat TM composited with topographic and climate variables. Therefore, it is suggested that the performance of the RF classifier on different satellite imagery data with different training sample strategies (balanced versus imbalanced) is different. 

With the SVM classifier, as shown in Figure 10 among the 42 classification results, the 8 highest accuracies belonged to the SVM classifier (SVM_set4, SVM_set5, SVM_set6, and SVM_set7). Particularly, the SVM classifier had the superior performance capability with small training sample sizes (set_1 and set_2 with approximately 50 pixels per class). SVM produced overall accuracies ranging from 93.76% to 93.96% (92.35% to 92.63%) for imbalanced (balanced) sub-datasets, compared to 89.80% to 92.06% (89.85% to 90.50%) and 90.71% to 92.34% (91.47% to 92.58%) for the kNN and RF classifiers, respectively. This is consistent with the study of Shao and Lunetta [62].

## 5. Conclusions

Classification of Sentinel-2 imagery (ten bands, 20 m) using three different machine learning algorithms were implemented, evaluated, and compared. Fourteen different sub-datasets, including balanced and imbalanced, with different training sample sizes from 50 to more than 1250 pixels/class were performed. All classification results (OA) were high, approximately ranging from 90% to 95%, with the SVM classifier on average producing the highest OA with the least sensitivity to training sample size, followed by the RF and kNN classifiers. The difference in OA between kNN and RF was large when the training sample size increased from sub-dataset-1 to sub-dataset-4 for both imbalanced and balanced cases; however, the difference between various training sample sizes of the SVM classifier was insignificant. For all three classifiers, when the training sample was large enough (greater than 750 pixels/class), with both imbalanced and balanced datasets (iset_5/bset_5, iset_6/bset_6, and iset_7/bset_7), the OA was approximately similar and high (over 93.85%). Furthermore, it is recommended that in land cover classification using remote sensing images and machine learning algorithms, the training sample size should represent approximately 0.25% of the total study area.

## Figures and Tables

**Table 1:** Spectral bands of the Sentinel-2 A satellite imagery.

Spectral Band	Center Wavelength (nm)	Band Width (nm)	Spatial Resolution (m)
Band 1	443	20	60
Band 2	490	65	10
Band 3	560	35	10
Band 4	665	30	10
Band 5	705	15	20
Band 6	740	15	20
Band 7	783	20	20
Band 8	842	115	10
Band 8a	865	20	20
Band 9	945	20	60
Band 10	1380	30	60
Band 11	1610	90	20
Band 12	2190	180	20

**Table 2:** Training and testing sample sizes used in this study.

Land Cover	Training (polygon/pixels)	Testing (pixels)
Residential	135/1410	625
Impervious surface	135/1645	427
Agriculture	135/2619	614
Bare land	135/1274	605
Forest	135/1267	629
Water	135/1704	628

**Table 3:** Samples data for training classification.

Imbalanced_data	iset_1	iset_2	iset_3	iset_4	iset_5	iset_6	iset_7
No. pixels	5%	10%	20%	40%	60%	80%	100%
**Balanced data**	**bset_1**	**bset_2**	**bset_3**	**bset_4**	**bset_5**	**bset_6**	**bset_7**
No. pixels	50	100	250	500	750	1000	1250

**Table 4:** The optimal parameters (C and γ) of different sub-datasets varied.

Imbalanced Dataset	γ	C	Balanced Dataset	γ	C
iset_1	0.03125	64	bset_1	0.03125	64
iset_2	0.5	32	bset_2	0.125	32
iset_3	0.03125	128	bset_3	0.125	64
iset_4	0.25	32	bset_4	0.125	64
iset_5	0.125	64	bset_5	0.125	128
iset_6	0.5	32	bset_6	0.25	64
iset_7	0.25	64	bset_7	0.25	128

**Figure A1:**
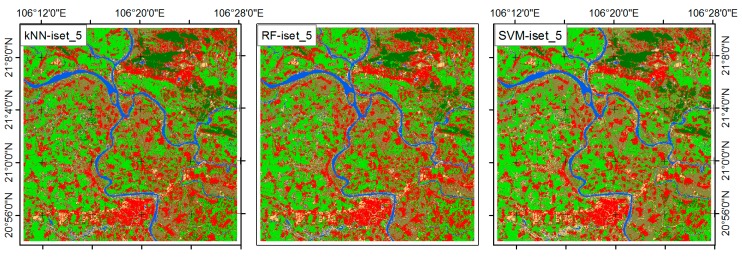

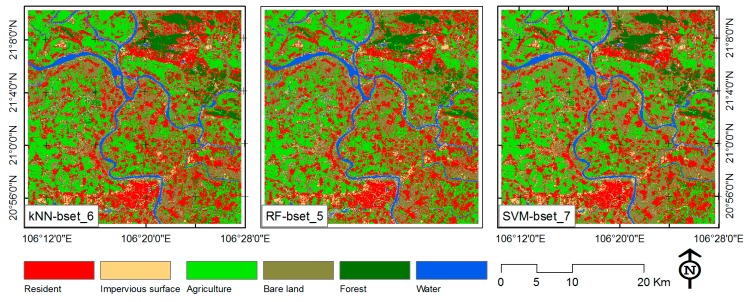
The classification results with the highest overall accuracy for each classifier.

## Appendix B

**Table A1:** Overall Accuracy and 95% confidence interval (error tolerance) of the probability estimate of all 42 classification results.

Classifier	set1	set2	set3	set4	set5	set6	set7
RF_iset	90.71 ± 1.0	92.34 ± 0.9	93.64 ± 0.8	93.97 ± 0.8	94.70 ± 0.7	94.32 ± 0.8	94.44 ± 0.8
RF_bset	91.47 ± 0.9	92.58 ± 0.9	92.60 ± 0.9	93.61 ± 0.8	94.59 ± 0.7	94.42 ± 0.8	94.37 ± 0.8
SVM_iset	93.96 ± 0.8	93.76 ± 0.8	94.33 ± 0.8	94.78 ± 0.7	95.32 ± 0.7	95.12 ± 0.7	95.07 ± 0.7
SVM_bset	92.63 ± 0.9	92.35 ± 0.9	93.71 ± 0.8	94.95 ± 0.7	95.10 ± 0.7	95.07 ± 0.7	95.29 ± 0.7
kNN_iset	89.80 ± 1.0	92.06 ± 0.9	92.60 ± 0.9	93.37 ± 0.8	94.59 ± 0.7	93.85 ± 0.8	94.13 ± 0.8
kNN_bset	89.85 ± 1.0	90.50 ± 1.0	91.81 ± 0.9	93.45 ± 0.8	93.96 ± 0.8	94.10 ± 0.8	94.02 ± 0.8
